# Utilizing the fecal microbiota to understand foal gut transitions from birth to weaning

**DOI:** 10.1371/journal.pone.0216211

**Published:** 2019-04-30

**Authors:** Ubaldo De La Torre, John D. Henderson, Kathleen L. Furtado, Madeleine Pedroja, O’Malley Elenamarie, Anthony Mora, Monica Y. Pechanec, Elizabeth A. Maga, Michael J. Mienaltowski

**Affiliations:** Department of Animal Science, University of California Davis, Davis, California, United States of America; Universite Paris-Sud, FRANCE

## Abstract

A healthy gastrointestinal (GI) tract with a properly established microbiota is necessary for a foal to develop into a healthy weanling. A foal’s health can be critically impacted by aberrations in the microbiome such as with diarrhea which can cause great morbidity and mortality in foals. In this study, we hypothesized that gut establishment in the foal transitioning from a diet of milk to a diet of grain, forage, and pasture would be detectable through analyses of the fecal microbiotas. Fecal samples from 37 sets of foals and mares were collected at multiple time points ranging from birth to weaning. Bacterial DNA was isolated from the samples, and the V4 domain of bacterial 16S rRNA genes were amplified via polymerase chain reaction. Next generation sequencing was then performed on the resulting amplicons, and analyses were performed to characterize the microbiome as well as the relative abundance of microbiota present. We found that bacterial population compositions followed a pattern throughout the early life of the foal in an age-dependent manner. As foals transitioned from milk consumption to a forage and grain diet, there were recognizable changes in fecal microbial compositions from initial populations predominant in the ability to metabolize milk to populations capable of utilizing fibrous plant material. We were also able to recognize differences in microbial populations amongst diarrheic foals as well as microbial population differences associated with differences in management styles between facilities. Future efforts will gauge the effects of lesser abundant bacterial populations that could also be essential to GI health, as well as to determine how associations between microbial population profiles and animal management practices can be used to inform strategies for improving upon the health and growth of horses overall.

## Introduction

A foal grows from about 10% of its mature body weight at birth to as much as 50% of that weight by the time of weaning [1]. As the foal grows, synchronization occurs between the changes in dietary needs, changes in type of food consumed (e.g., changes in mare’s milk composition, introduction of creep feeding to transition to a solid diet), and shifts in the gut microbiota to bacterial populations that can more efficiently utilize the diet provided. Due to fairly recent advancements in ‘omic’ technologies, the importance of the microbiota on health is being realized because of the sudden increase of available information on gut microbiota composition and functions. The gut microbiota may even be seen as an organ system in the host given the important roles it plays in processing ingested organic matter [2]. The health of the host, or in this case the foal, is dependent upon these microbes and can be impacted by perturbances to the microbiota such as those caused by infectious diseases or antibiotic treatment.

Naturally, the mare provides some defense to the foal via immunoglobulins in colostrum and milk and levels of innate anti-microbial molecules like lysozyme in the milk [3]. These initial contacts with the mare may begin to provide the foal with early colonizing microbes. Studies have begun to understand which microbial populations comprise healthy and unhealthy gut microbiomes and how they may change once the foal no longer relies on the mare for food [4–7]. Since there are many risks to the well-being of a neonate’s GI health, developing methods to track and assess GI health would be advantageous. Bacterial community structures in the foal have been monitored using ribosomal intergenic spacer analysis which is a “fingerprint” of gut microbiota diversity but does not specifically delineate composition details [8]. Others have tried to specifically identify populations by culturing specific species from foals in the first 5 weeks of life, but only those populations capable of culture were studied [9]. Infectious bacterial populations have been specifically interrogated via development of microbial species-specific diagnostic tools [10–12].

This study utilizes Next-Generation Sequencing (NGS) technology to continue comprehensively analyzing gut microbial composition and establishment in foals as represented by fecal samples. We hypothesized that differences in gut establishment by age and by diarrhea status would be detectable in analyses of the fecal microbiotas of foals from birth to weaning. Here we report on identity and quantity of microbial species to understand the colonization of the gut microbiota in foals leading to the establishment of a reference catalog of bacteria present in the feces of foals that are either healthy or ill. The study affirms that NGS can be used as a tool to track and predict the GI health status of a foal from birth to weaning. Improved understanding of foal gut microbiota in various health states will aid in optimizing care of foals from postnatal to weaning stages.

## Materials and methods

### Horses and sample collection

The UC Davis IACUC approved our study (#18998): “Characterization of the Gut Microbiota of Foals from Birth to Weaning.” Voluntarily-voided fecal samples were collected from 37 sets of foals and mares from several breeds at three different farm locations (S1 Table). A majority of horses were either Thoroughbreds or American Quarter Horses. Management for the farms had similarities and differences; mares were fed combinations of supplemental grain and hay with consideration for pasture access at two farms (S2 Table) to provide nutrient requirements as recommended by the Committee on Nutrient Requirements of Horses (National Research Council Board on Agriculture and Natural Resources, National Academy of Sciences) [13, 14]. Samples were collected from foals at days 1, 7, 28, 60, and at weaning; samples were collected from mares at day 1 of the birth of the foals (S1 Table). The fecal samples were stored in polypropylene tubes at -20°C until DNA isolation was performed at about 30 days post-collection.

### Bacterial DNA isolation, library preparation, and sequencing

One hundred sixty-three fecal samples were thawed and PCR-quality DNA was isolated based upon manufacturer’s instructions (ZR Fecal DNA Kit, Zymo Research) [15] with adaptations to increase centrifugation speeds. DNA concentration was determined using a NanoDrop UV spectrophotometer (ThermoFisher Scientific). The V4 domain of bacterial 16S rRNA genes was then amplified to generate libraries bar-coded by sample. Primers F515 (forward: 5’-GTGCCAGCMGCCGCGGTAA-3’) and R806 (5’-GGACTACHVGGGTWTCTAAT-3’) were used to amplify the V4 domain and included a unique barcode on each forward primer [16]. PCR was performed in triplicates for 25-ul reactions using GoTaq 2X Green Master Mix (ProMega) and programmed to follow: 1 initial step at 94°C for 3 min, followed by 35 cycles of 94°C for 45 sec, 50°C for 1 min, and 72°C for 90 sec, ending with a final extension at 72°C for 10 min. PCR amplification success was examined using agarose gel electrophoresis after triplicates were combined for each sample. Purification of PCR products was performed using QIAGEN’s PCR Purification Kit. Combined barcoded libraries were submitted to the University of California Davis Genome Center DNA Technologies Core for 250bp paired-end sequencing using the Illumina MiSeq platform. Raw sequence data are freely available at the Sequence Read Archive (SRA): Bio Project PRJNA475435, BioSample Accession SAMN09389119.

### Microbiota analyses

DNA sequences from NGS were initially processed using QIIME 1.9.1 (Quantitative Insights Into Microbial Ecology) open-source software [17]. All sequences were demultiplexed, filtered, and assigned an operational taxonomic unit (OTU) using the Greengenes database (v13.8) at 97% identity in order to perform further diversity analyses. To filter sequences, a minimal fraction threshold of 0.005% of reads was used in QIIME. After filtering, samples were rarified to 3,590 sequences from each sample based on the samples with the lowest number of reads, and thus allowing for the incorporation of all samples in downstream analyses. QIIME was used to create rarefaction curves, taxonomical bar plots, and PCA (Principal Component Analysis) plots. OTU tables produced by QIIME were extracted and inputted into LEfSe (LDA Effect Size) and STAMP (Statistical Analysis of Metagenomic Profiles) software [18, 19] with data examined across different hierarchical levels starting from level 2 (Phylum) to level 6 (Genus). For LEfSe, differentially abundant features were determined using a non-parametric factorial Kruskal-Wallis (KW) rank-sum test to categorize statistical differences seen between two or more groups. After identifying statistical differences between groups, additional tests were done using the Wilcoxon rank-sum test and Linear Discriminant Analysis in order to normalize samples and assess if the differences were consistent with biological relevancies [19]. Using LEfSe, comparisons were made between two groups at any one time (e.g., Day 1 vs. Day 60, Farm A vs, Farm B, etc.). For STAMP, OTUs were assigned to different subsystems or biological pathways. Statistical tests were run on the samples at various hierarchical levels using effect sizes and confidence intervals to assess the biological importance of bacterial communities’ interactions with foals. A taxonomic abundance data file was submitted into STAMP along with a metadata file containing information such as foal/mare identity and time point. Default parameters were used with the addition of a Bonferroni multiple test correction. Data outputs from STAMP were input into R-studio; statistical outputs in a multiple group statistics table created by STAMP were inputted to create dendritic heatmaps [20]. Mean relative frequencies of each OTU in each fecal sample allowed for visualization of relatedness amongst the samples along with the abundance of specific bacteria in different groups.

## Results and discussion

### Diversity analysis

**Fig 1:**
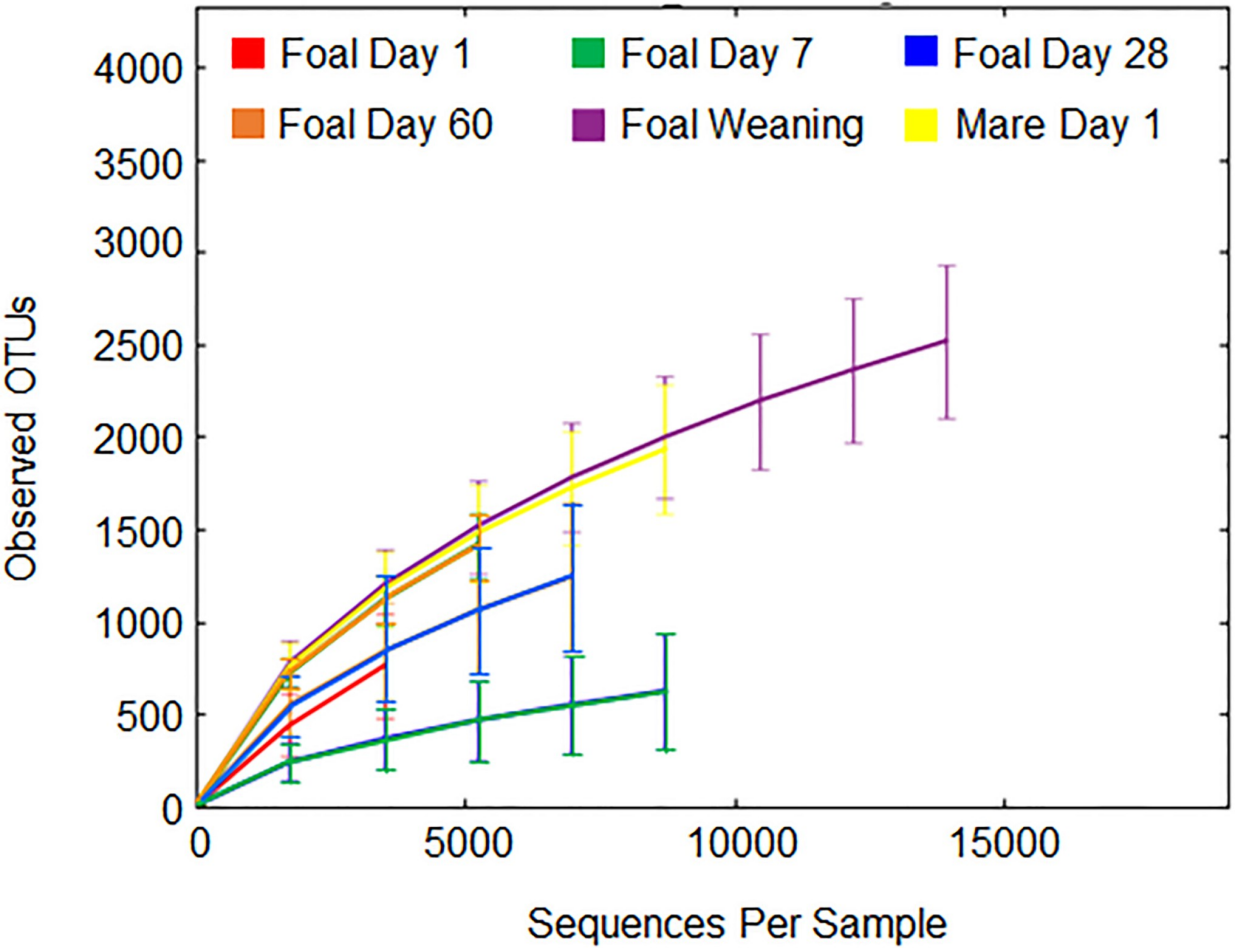
Rarefaction curve compares diversity and depth coverage amongst samples segregated by time points. As a general trend, the greatest depth of coverage by sample was seen for foals at Day 60 and weaning as well as in adult mares. This curve was used to determine the number of sequence reads to use as a threshold to capture more fecal samples in the study.

From the 163 samples, 11,793,830 reads were generated. In QIIME, sequence reads were segregated by sample, which represents a horse (mare or foal) at a time point after birth. For 3,102,798 reads, OTUs were picked based on sequence similarity using the Greengenes reference library; after filtering for a minimum of two counts per OTU, 2,447,151 reads had OTUs picked for a mean of 15,103 counts per sample (Table 1). A rarefaction curve was generated to compare level of diversity and depth of coverage by time point (Fig 1). Numbers of diverse OTU assignments were similar amongst Day 60 foals, weaned foals, and mares. The least diversity was seen at Day 7, and coverage depth was smallest for Day 1 foal fecal samples. The lack of a plateau on the rarefaction curve graphing taxonomic diversity versus sequencing depth demonstrated that saturation of observed OTUs was not met; however, increasing the threshold for minimal reads picked for OTUs above 3590 reads would have reduced the number of horses represented. Differences in alpha diversity most often demonstrated greater alpha diversity by age (S3 Table).

**Table 1:** Sequencing Results.

Samples	163
Reads generated	11,793,830
QIIME unfiltered counted reads	3,102,798
QIIME filtered counted reads	2,447,151
Mean counts per sample	15,013
Standard deviation mapped reads per sample	6,734

**Fig 2:**
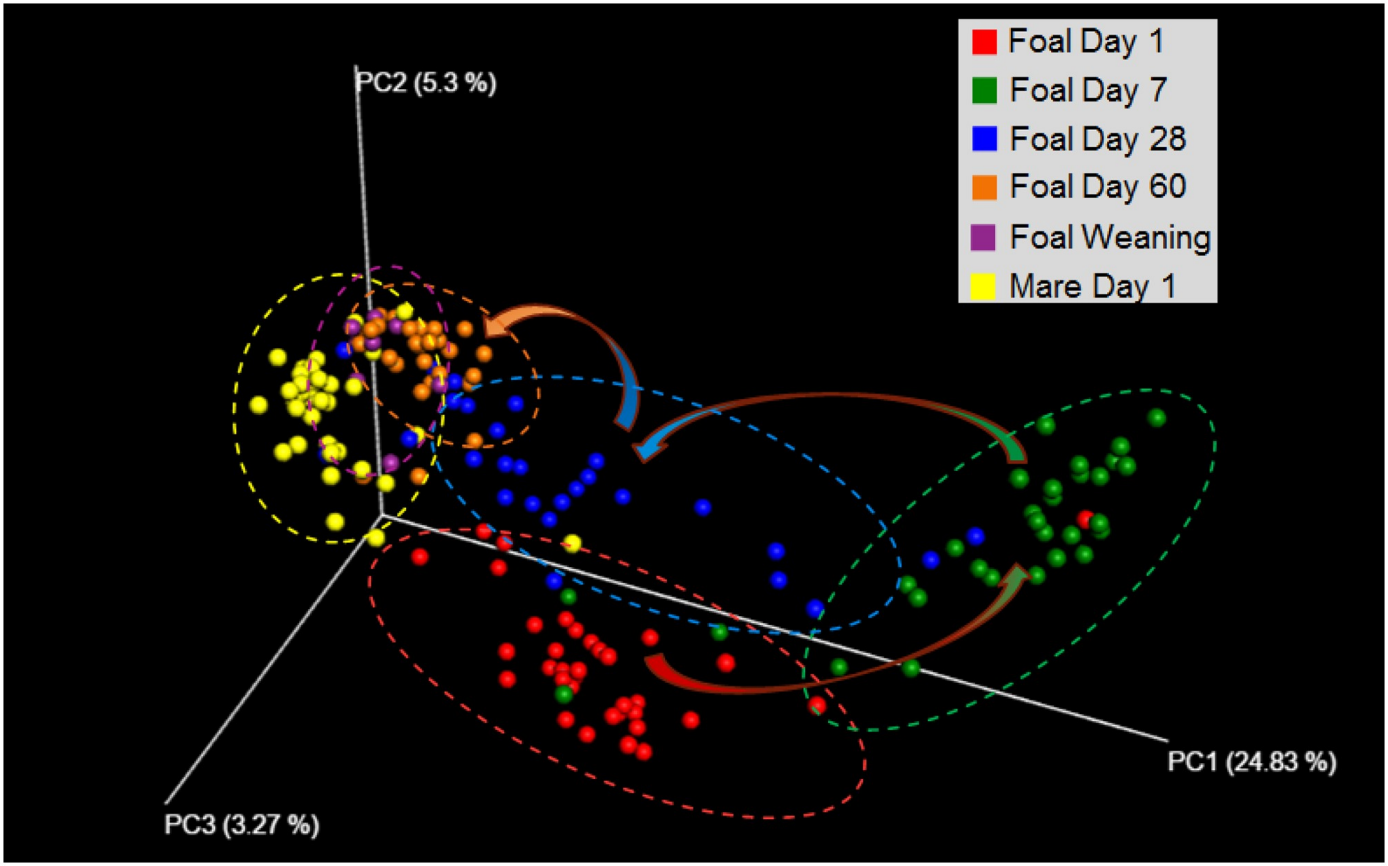
PCA demonstrates how globally foal microbiota changes toward adult microbiota. A principal component analysis of bacterial populations found from sequence data shows that the microbiota transitions toward the adult GI microbiota. Foal samples at Days 1, 7, 28, 60, and weaning, respectively, cluster by day with Day 60 and weaning samples overlapping with the adult mare. Loose clusters represent periods of variability. Segregation of samples by farm can be found in S1 Fig.

**Fig 3:**
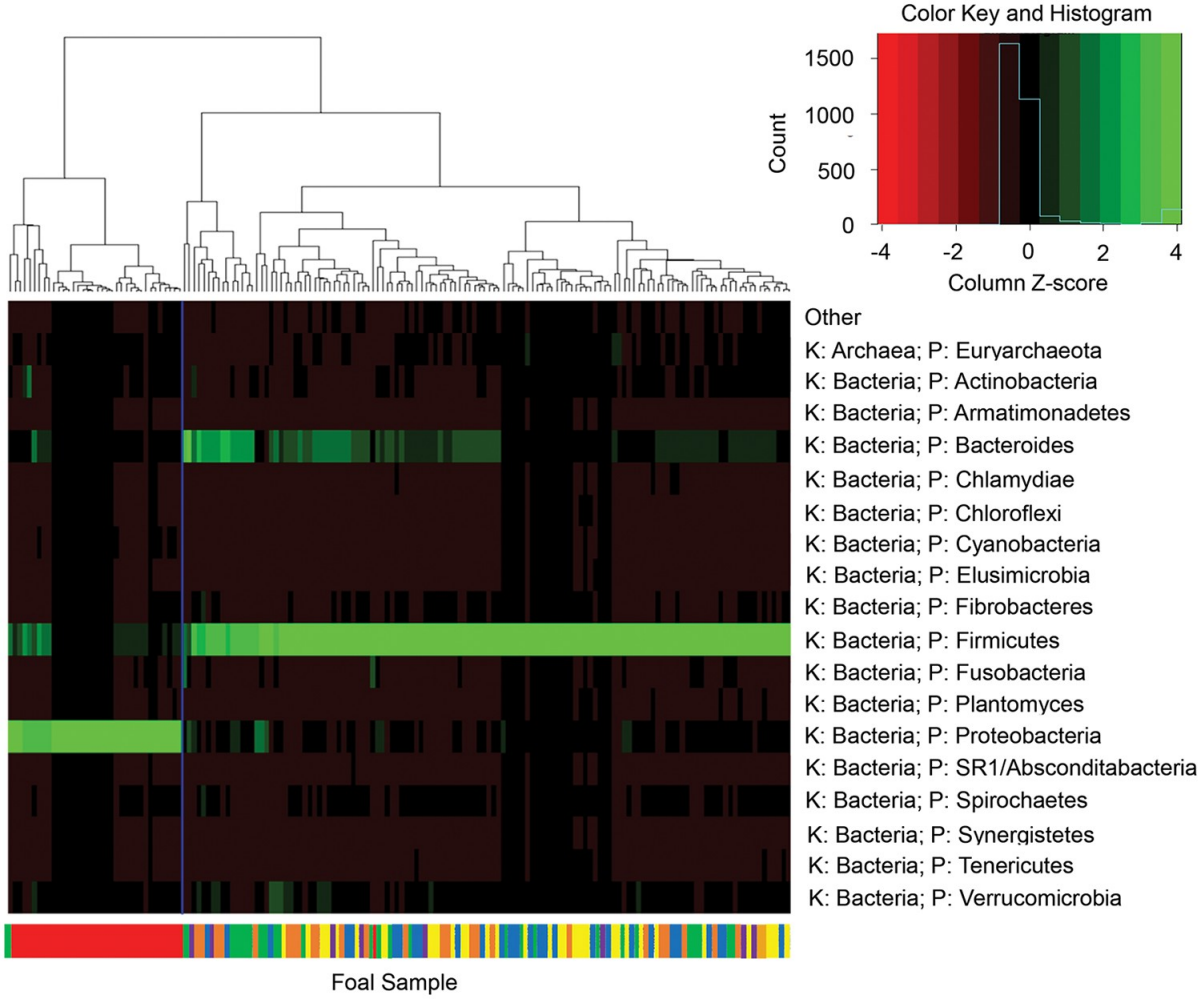
A dendritic plot and heatmap of the phyla present within each sample’s sequencing data demonstrate how individual fecal samples cluster together. From the sequence data, the most obvious finding in the heatmap is that Day 1 samples all have bacteria of the Phylum *Proteobacteria* in common, while samples outside of Day 1 all share bacteria from the Phylum *Firmicutes*. At the bottom of the heatmap is a color code of the samples in which the colors designated for each day are the same as Fig 2. In this heatmap, Day 1 foal samples cluster together; samples from other days cluster with each other.

Microbial populations from all fecal samples sequenced were subjected beta diversity analysis. An unweighted Unifrac PCA is represented in Fig 2. The compositions of bacterial populations for the samples follow a pattern throughout the early life of the foal. Day 1 and 7 samples, respectively, cluster according to their time points, regardless of farm. There is a transition of samples up to and through Day 28. Then Day 60, weaning, and adult samples (mare) cluster together. These data indicate that by Day 60 the foal’s GI microbiota has been established to include the bacteria necessary for the digestion of the roughage typically found in the mature horse diet, much like what had been shown in a study of Quarter Horses in Canada [5]. A dendritic heatmap of phyla level microbial population data shows a significantly high abundance of *Proteobacteria*, mainly of the *Acinetobacter* genus, in Day 1 foals compared to the other age groups (Fig 3). *Proteobacteria* continued to be present in the Day 7 samples and lowered significantly by Day 28. From age groups Day 7 to day of weaning and mares, abundance of *Firmicutes* followed by *Bacteroidetes* was observed throughout the samples with a similarity between the age groups up to the family level. Other studies have also shown that *Firmicutes* are the most abundant phylum in horses [5, 21]. When examining samples for each farm, farm-dependent clustering dependent on farm location was not observed in the dendritic heatmap; however, farm-to-farm comparisons were further explored using LDA Effect Size (LEfSe) analysis.

**Fig 4:**
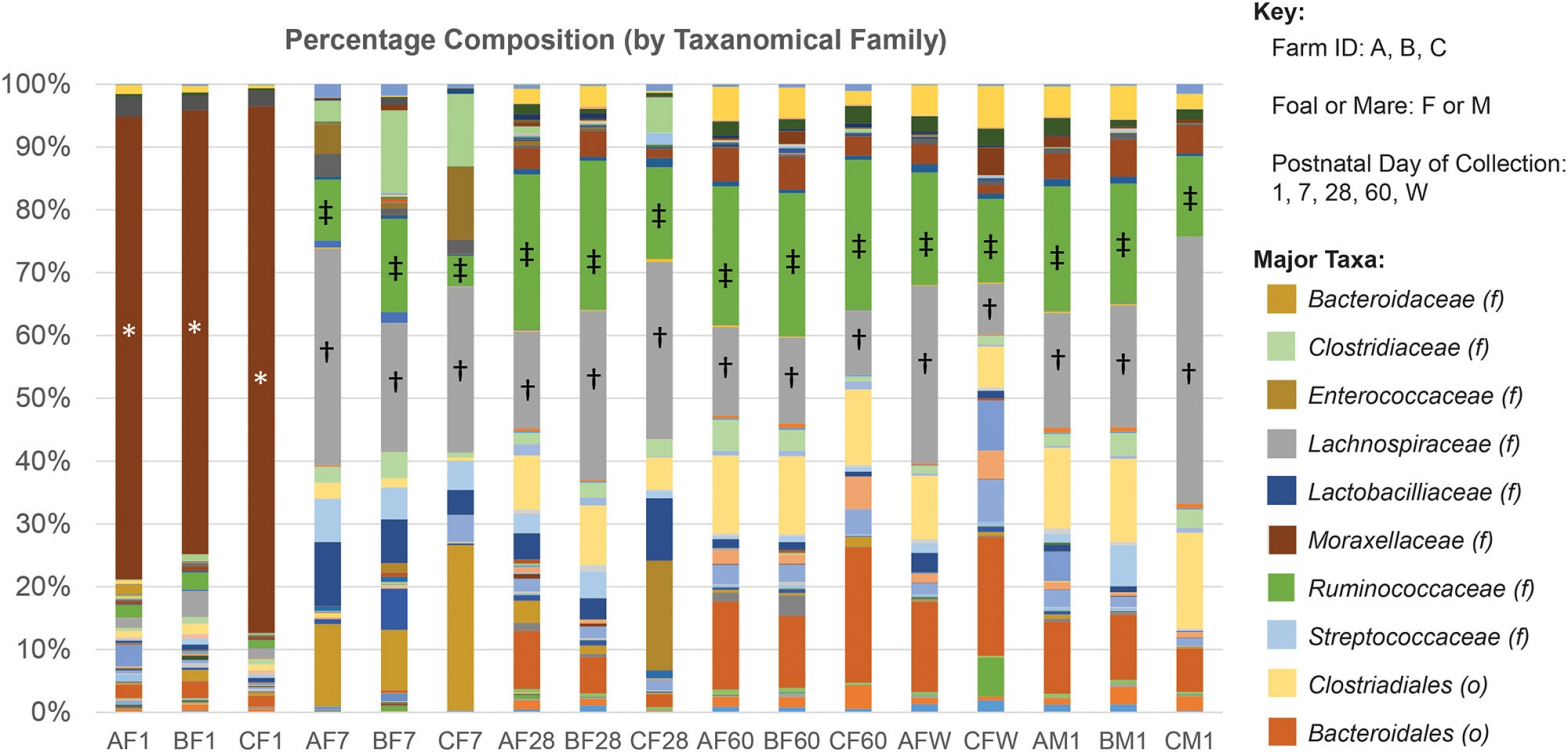
QIIME’s taxonomical plots can be used to compare and contrast across farm, age, and mare or foal status. From the preliminary sequence data, certain trends can be seen. For example, in the first day of life fecal samples contain higher levels of bacteria from the *Moraxellaceae* family (*), commonly found in soil. By Day 7, there is a great deal of variation. Two common populations in foals from Day 7 until weaning, and in mares, include bacteria from families *Lachnospiraceae* (†) and *Ruminococcaceae* (‡) necessary for breakdown of complex carbohydrates like with grass diets.

Microbial populations were then examined in taxa bar plots; we detected differences in microbial populations by age and farm (Fig 4, S4 Table). *Pseudomonas* and *Acinetobacter* of the class *Proteobacteria*, including from family *Moraxellaceae*, were highly present in Day 1 fecal samples while *Enterobacteriaceae*, *Fusobacterium*, *Erysipelotrichaceae*, *Peptostreptococcaceae*, and *Bacteroides* were highly present in Day 7 fecal samples. A newborn’s gut represents a positive redox potential allowing for the growth of benefitting facultative anaerobes [22]. *Pseudomonas'* capability to rapidly multiply and its opportunistic nutritional strategy allow it to flourish in a foal’s initial gut colonization [23]. Along with *Pseudomonas*, other facultative anaerobes such as *Fusobacterium* and *Peptostreptococcaceae* can begin to predominate the intestinal microbiota [24]. Notably, previous studies have shown that numbers of *Bacteroides* rise sharply after the introduction of solid food [25]. At a week of age, we expect the foals to be progressively exposed to solid food through contact with their mother’s concentrate and forage corresponding to the higher abundance of these bacteria observed in day 7 foals [24]. Greater levels of Enterobacteriaceae at Day 7 are likely the result of exposure of the foal to the mare’s feces and copraphagic events affecting the foal’s microbiota [26, 27]. It should also be noted that two common microbial families in foals from Day 7 until weaning, and in mares, were *Lachnospiraceae* and *Ruminococcaceae* necessary for breakdown of complex carbohydrates like with grass diets [28]. Quercia *et al*. theorized bacteria from these families from the mares help with colonization of the foal GI [7].

#### LEfSe analysis comparing populations

LDA Effect Size (LEfSe) was used to classify differentially abundant microbiota features between subgroups of foals. After performing a KW sum-rank test to categorize statistically different levels of bacteria between groups, differentially abundant populations were found when comparing diarrheic and non-diarrheic samples, when comparing samples at different postnatal time points, and when comparing between the horses on the two major farms examined.

**Fig 5:**
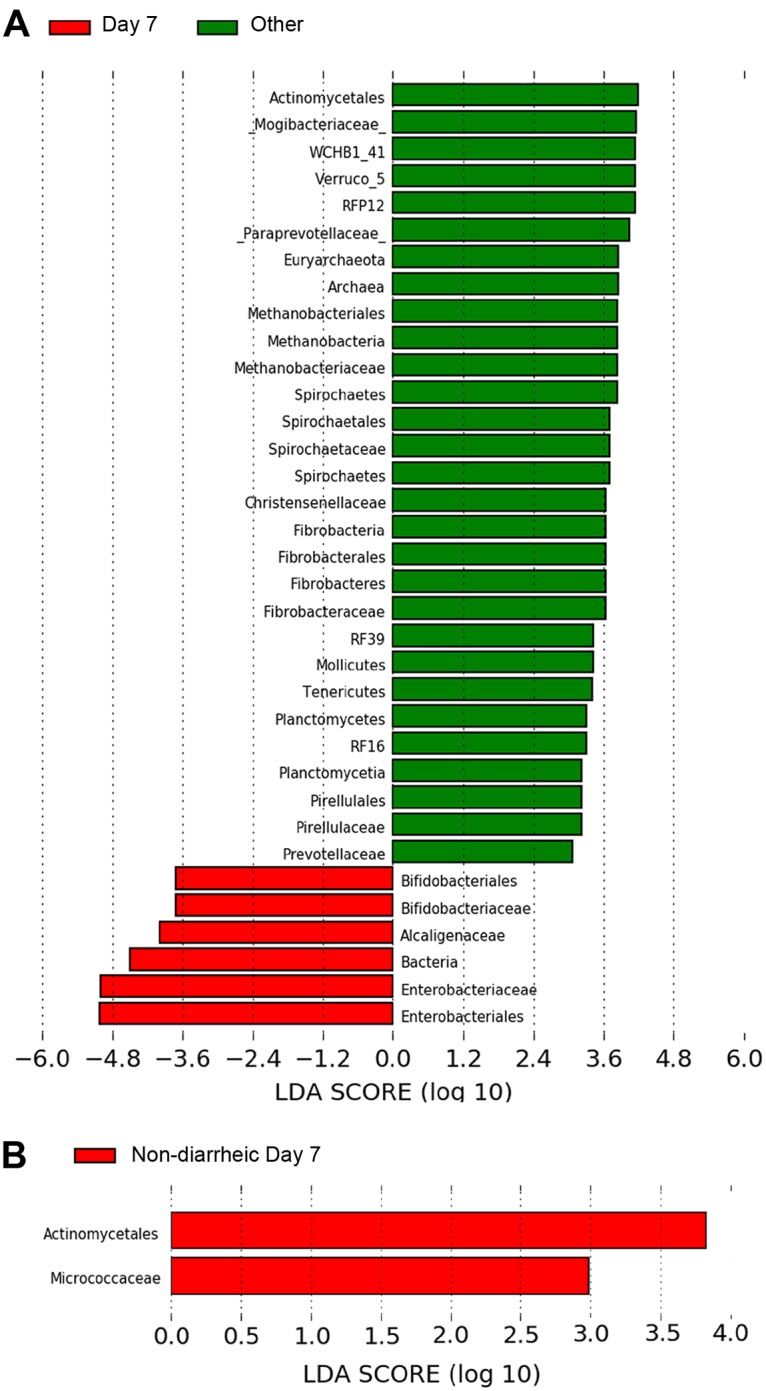
LEfSe distinguishes difference in relative abundance of bacterial families in diarrheic samples at Day 7. **(A)** Significant differences in microbial populations were examined for Day 7 diarrhea samples versus all other diarrhea samples. **(B)** Differences between microbial populations of Day 7 diarrhea samples versus non-diarrheic samples were also considered. Significant differences were found by Kruskal-Wallis (KW) rank-sum test, and reported in log 10 scale.

Ten of the 163 fecal samples (or 6.1%) in this study were diarrheic samples, representing incidence of diarrhea in eight foals (19.0%): one Day 1, four Day 7, two Day 28, two Day 60, and one at weaning. It should be noted that none of these foals had a failure of passive transfer of colostral immunoglobulins. Day 7 diarrheic samples were compared collectively to diarrheic samples at Days 1, 28, 60, and weaning (Fig 5A). Certain bacterial populations were greater in Day 7 relative to the other diarrheic samples. For example, for Day 7 diarrheic samples there was increased abundance of bacteria from the family *Enterobacteriaceae* including facultative anaerobes taking advantage of a newborn’s intestinal aerobic environment [29] and possibly representative of *Salmonella* species and *Escherichia coli*, as well as increased microbes from *Alcaligenceae*—though still <0.1%—which could represent the genus *Sutterella* which is linked to acute hemorrhagic diarrhea and inflammatory bowel disease [30]. Additionally, increased amounts of *Bifidobacteriaceae* bacteria were also present, likely representing the greater dependence of the Day 7 foal on milk relative to the older postnatal time points included in this comparison since microbes in this family are known to utilize lactose and milk oligosaccharides. Examination of some of the species seen in the other diarrheic samples could be indicative of dysbioses or just true differences by age such as *Spirochaetes* found in relatively higher numbers in healthy horses [31–33]. Compared to diarrheic Day 7 foal samples, Day 7 non-diarrheic samples were significantly enriched in order *Actinomycetales* especially the family *Micrococcaceae* (Fig 5B). *Micrococcaceae* and *Actinomycetales* have yet to be studied in detail within equine. In humans, members of the *Actinomycetales* order are associated with a healthy GI microbiota including the prevention of diarrhea, though *Micrococcaceae* are noted to be commensals [34, 35].

**Fig 6:**
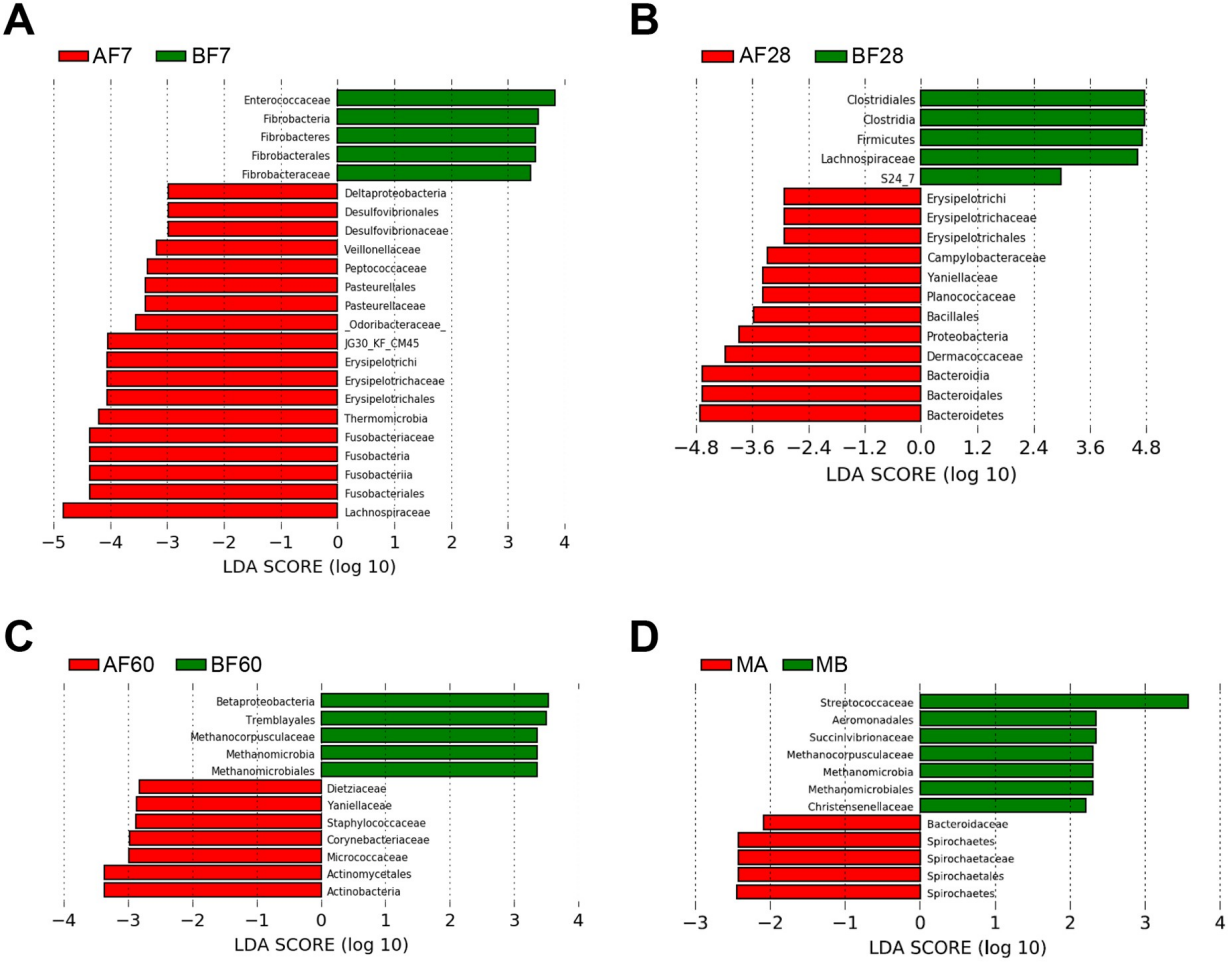
LEfSe distinguishes which bacterial populations are relatively abundant between healthy samples at Farm A and Farm B at different time points. Specifically, we examined **(A)** Day 7, **(B)** Day 28, **(C)** Day 60, and **(D)** mare samples at Day 1. Significant differences were found by Kruskal-Wallis (KW) rank-sum test, and reported in log 10 scale.

Comparisons were also made with LEfSe between the two major farms at Days 7, 28, and 60. At Day 7, there were several microbial populations that were enriched in Farm A foals; these included *Deltaproteobacteria*, *Desulfovibrionaceae*, *Veillonellaceae*, *Peptococcaceae*, *Pasteurellaceae*, *Odoribacteraceae*, *Erysipelotrichaceae*, *Thermomicrobia*, *Fusobacteriaceae*, *and Lachnospiraceae* (Fig 6A). Interestingly, bacteria from *Deltaproteobacteria*, *Desulfovibrionaceae*, *and Erysipelotrichaceae* are found in greater abundance in high fat diets like with milk consumption [36, 37]; thus, as Farm B was exclusively Thoroughbred, perhaps there was more milk fat in the mare’s milk of the several breeds at Farm A. Moreover, microbes from *Odoribacteraceae* and *Lachnospiraceae* have been found to contribute to the production of short chain fatty acids, particularly butyrate which is used as an energy source for gut epithelial cells [38] and aids in combatting against GI disorders, such as *Clostridial difficile* infection [39]. Farm B Day 7 foals were significantly more abundant in *Enterococcaceae* and *Fibrobacteraceae*. Studies have discussed the initial colonization of the neonatal intestinal microbiome containing *Enterococcaceae* before rapidly decreasing as *Firmicutes* begin to become dominant [40]. In the family *Fibrobacteraceae*, there is common bacteria, *Fibrobacter*, known to digest fiber which may be the result of the foal’s exposure to its new local environment [41].

In comparing the microbial populations of fecal samples at Day 28, there were proportional differences between classes of *Firmicutes*, *Actinobacteria*, and *Bacteroidetes*, and *Proteobacteria* (Fig 6B). The 4 phyla are commonly found to dominate the gut microbiome. Samples from Farm A had higher relative abundances of *Actinobacteria*, *Bacteroidetes*, and *Proteobacteria* than those of Farm B. The presence of certain subclasses of *Actinobacteria* is an indicator of a healthy gut given their association with immune response modulation to combat pathogens [42]. *Bacteroidetes* are associated with health benefits through the creation of short chain fatty acids via the digestion of complex sugars and proteins [43]. Microbes from *Proteobacteria*, *Campylobacteraceae*, and *Erysipelotrichaceae* were found in greater abundance in fecal samples from Farm A. *Campylobacteraceae* contain genera which are associated with equine GI disease [44]. Through examination of the *Firmicutes* phylum, Farm A contained higher levels of *Bacillales* and *Erysipelotrichi*, while Farm B contained higher levels of *Clostridia* including *Clostridiales*, and *Lachnospiraceae*. *Bacillales* and *Erysipelotrichi* have been associated to increase with a high-fat diet [45, 46]; perhaps at Day 28, Farm A’s foals were still relying more upon mare’s milk. Commensal *Clostridia* are essential for proper maintenance of the gut and participate in several processes including physiology, metabolism, and immune responses [47]. However, it has been proposed that high levels of *Clostridiales* contribute to inflammatory bowel syndrome symptoms through the increased concentrations of intestinal butyrate being produced [48].

For fecal samples from 60-day-old foals, Farm A contained a greater abundance of classes *Corynebacteriales (Dietziaceae* and *Corynebacteriaceae)* and *Micrococcales* (*Micrococcaceae*) from the phylum *Actinobacteria* and *Bacillales* (*Staphylococcaceae*) from the phylum *Firmicutes* (Fig 6C). Species of the family *Dietziaceae* have been suggested to have a novel fatty acid biosynthesis system while high levels of *Corynebacteriaceae* have been associated with breast milk bacterial communities [49, 50]. *Staphylococcaceae* species are capable of causing infections in multiple animals including equine, bovine, and swine, while species in the order *Micrococcales* are associated with soil-born decaying plants [34]. Farm B represented increased levels of *Euryarchaeota* and *Proteobacteria*. Microbes from the Phylum *Euryarcheota* include methanogens and sulfide-reducers, suggesting that at sixty days of age horses from Farm B were more likely utilizing pasture than a forage-grain diet, relative to Farm A [51]. This is indeed the case as the horses are kept on pasture with hay forage offered for Farm B while horses in Farm A were fed hay with very limited pasture. In comparing the bacteria in the feces of foals from Farms A and B, differences in fecal microbiotas for the horses on each farm could be associated with differences in management. However, more thorough and better controlled studies will need to be done to better resolve such relationships.

Microbial populations of fecal samples from mares at the day of their foals’ birth were also compared on Farms A and B (Fig 6D). As mentioned above, observations from these differences could lead to further better-controlled studies to resolve relationships between these microbial populations and farm-to-farm difference. There were more microbes from the families *Bacteroidaceae* and *Spirochaetaceae* in the feces of mares from Farm A, while there were more microbes from the families *Streptococcaceae*, *Succinivibrionaceae*, *Methanocorpusculaceae*, and *Christensenellaceae* in the feces of mares from Farm B. Microbes from the families *Bacteroidaceae* and *Spirochaetaceae* contribute to the core composition of the bacteria in the equine large intestine [32]. Others have noted that relative decreases in *Bacteroidaceae* have been observed in animals that are stressed [6, 52]. Decreased relative levels of *Bacteroidaceae* along increased relative abundance of bacteria from the family *Succinivibrionaceae*, which have been found to increase with stress in horses [53, 54], proffer another study for further consideration of diet, stress, and parturition, much like Mach *et al*. did comparing salivary cortisol levels during weaning [6]. Some bacterial species in the family *Christensenellaceae* have been found in leaner animals; as mares in Farm B were exclusively Thoroughbreds, the differences in abundance of bacteria from the family could be due to breed differences in prevalence of obesity and Equine Metabolic Syndrome [55, 56]. A comparison between the fecal samples of the mares also seems to offer insight into possible associations with differences in conditions at each farm.

**Fig 7:**
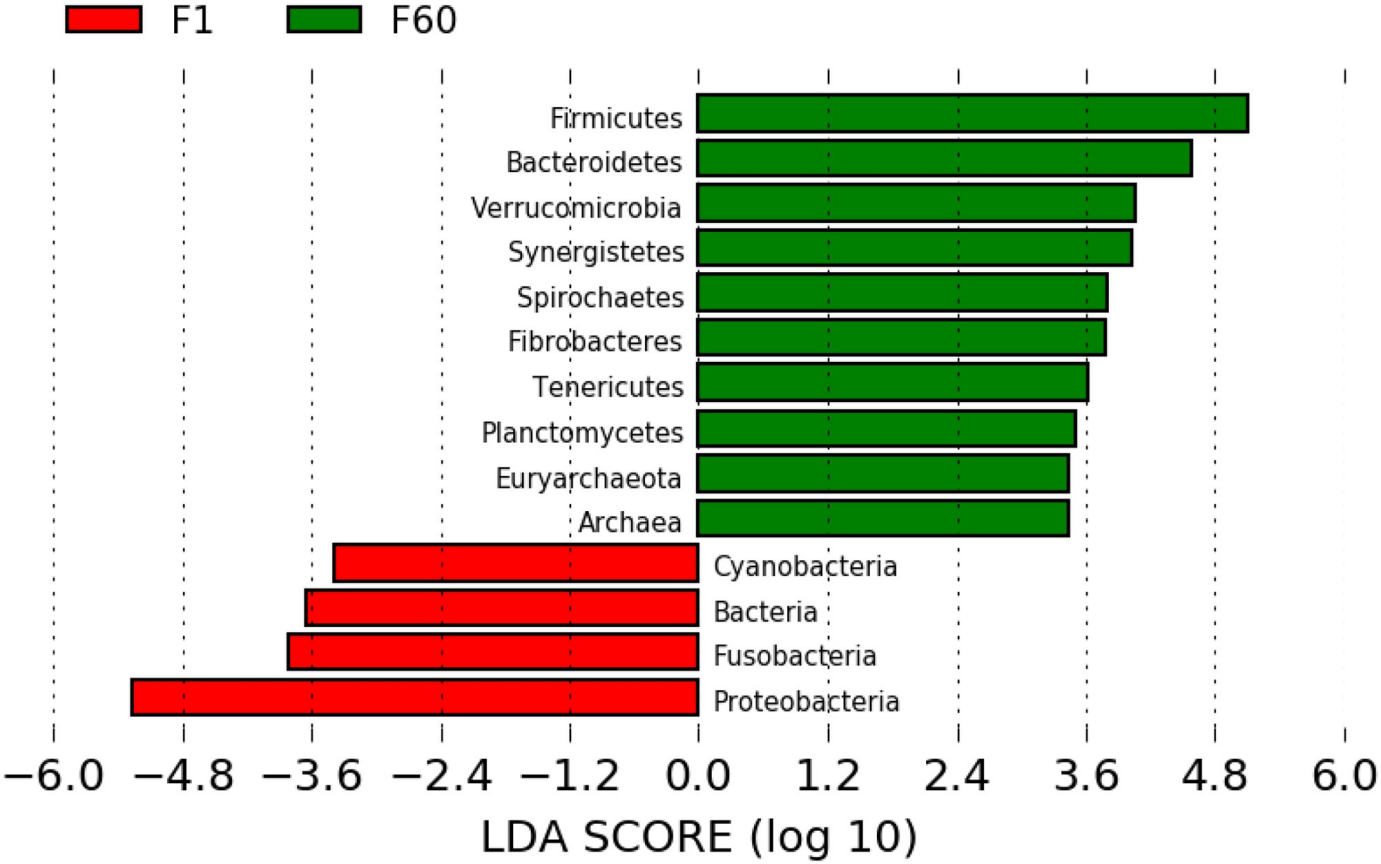
LEfSe distinguishes which bacterial populations are relatively abundant between healthy samples at Day 1 vs. Day 60. Significant differences in microbial populations were found when comparing fecal samples of foals in the first day of life which rely upon mare’s milk with fecal samples from the same foals at Day 60 that also consume grains, pasture, and forage. Significant differences were found by Kruskal-Wallis (KW) rank-sum test, and reported in log 10 scale.

In addition, specific comparisons were made between the fecal samples of foals at Day 1 and Day 60 which was a time point in this study in which the gut microbiome was closely similar to that of the fecal microbiota of adult horses (Fig 7). At Day 1, *Cyanobacteria*, *Fusobacteria*, and *Proteobacteria* were significantly more abundant. Day 60 samples were more abundant for *Firmicutes*, *Bacteroidetes*, *Verrucomicrobia*, *Synergistetes*, *Spirochaetes*, *Fibrobacteres*, *Tenericutes*, *Planctomycetes*, and *Euryarchaeota*. *Firmicutes* are involved in assisting with the digestion of insoluble fiber and hindgut fermentation [57]. *Bacteroidetes* are also known to assist in hindgut fermentation, but to a lesser degree than *Firmicutes* [57]. *Verrucomicrobia* also play a role in hindgut fermentation and contains bacteria in its phylum known to be associated with healthier metabolic status in humans [58, 59]. However, *Verrucomicrobia* have been noted to increase with chronic laminitis in horses [59]. Not much is currently known about the phylum *Synergistetes*, but one study has described a positive correlation between the amount of *Synergistetes* and the production of anti-inflammatory antibodies [60]. *Fibrobacteres* has been found in the large intestine of adult horses and may play a role in the digestion of cellulose and other fibers [32]. In mouse, the amount of *Tenericutes* in the gut microbiome decreases with an increase of fat in the diet which is not typical of an adult horse diet [61].

#### STAMP analysis

**Fig 8:**
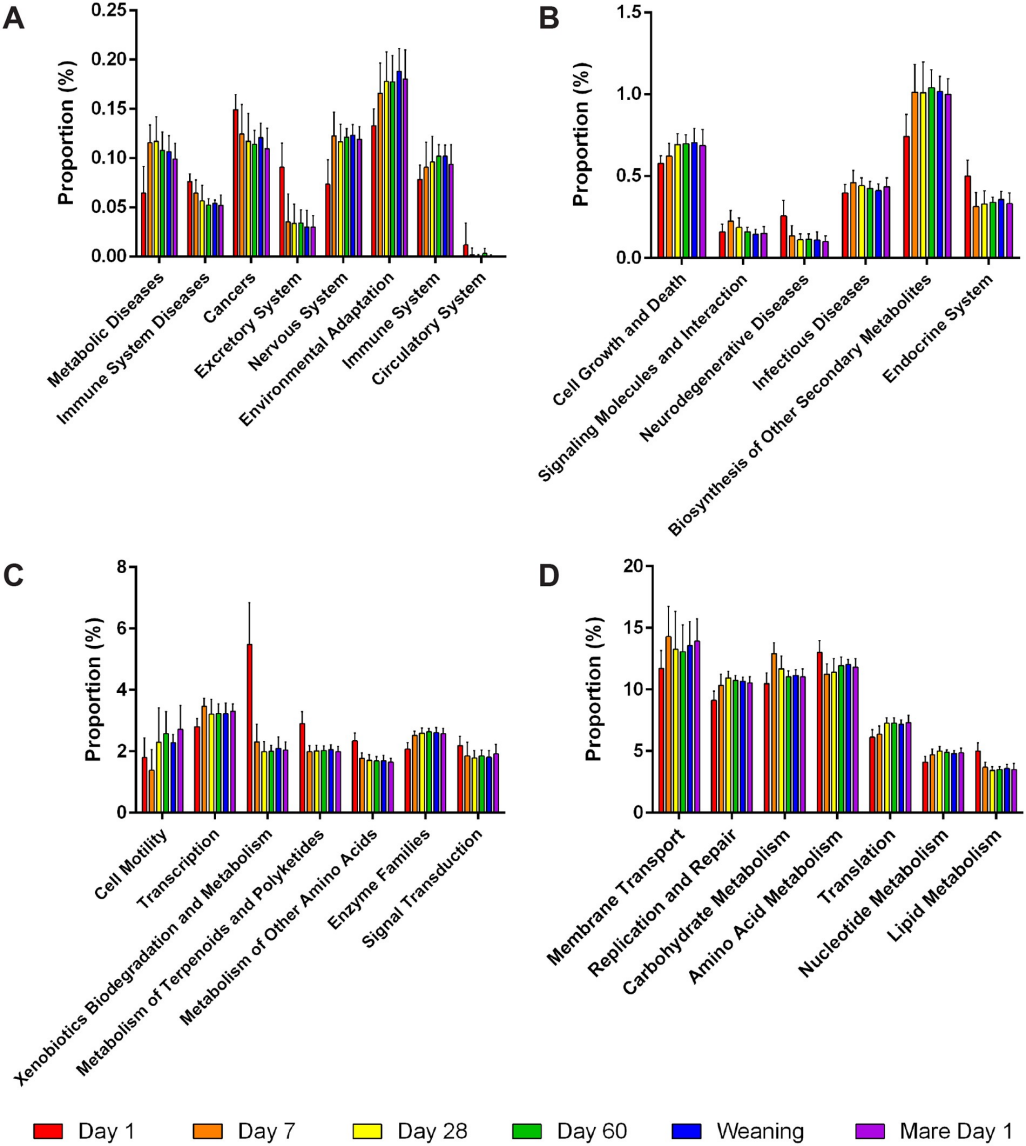
STAMP (STatistical Analysis of Metagenomic Profiles) was used to characterize functional roles of microbial populations in host biology by age. After Bonferroni correction, twenty-eight categories were considered to have differed significantly at some point in the transition of microbial populations from birth to weaning. In the above panels, the categories are divided by proportion ranges of **(A)** 0.0%—0.25%, **(B)** 0.25% -1.5%, **(C)** 1.5%—8.0%, and **(D)** 5%—20%. Bars are represented as mean ± standard deviation.

Statistical analysis of taxonomic and functional profiles (STAMP) was used to characterize functional roles of microbial populations in host biology. At the phylum level after Bonferroni correction, 28 categories were considered to have differed significantly at some point in the transition of microbial populations from birth to weaning (Fig 8). Notably, as pertaining to the foal’s diet over time, categories like metabolic diseases (Fig 8A), biosynthesis of other secondary metabolites (Fig 8B), metabolism of amino acids (Fig 8C), carbohydrate metabolism (Fig 8D), amino acid metabolism (Fig 8D), nucleotide metabolism (Fig 8D), and lipid metabolism (Fig 8D) demonstrate proportional differences representative of the digestion of milk-rich nutrients in a neonatal diet transitioning toward a complex carbohydrate-rich grain and forage diet. Also interesting was the dramatic difference between microbial populations typically characterized in responding to exposure to foreign compounds like xenobiotics, as well as polyketides (e.g., naturally occurring insecticides, antibiotics, antifungals, etc.) and terpenoids found in plants, possibly in response to a foal’s first exposure to its environment after parturition. Some of these xenobiotic exposures are found when STAMP is used to consider differences at class level, *e*.*g*., styrene degradation, atrazine degradation, naphthalene degradation, etc. (S5 and S6 Tables).

#### Considering storage and comparisons

In this study, fecal samples were stored at -20°C. Many studies have suggested that storing samples in liquid nitrogen and/or -80°C could be considered the gold standard [62–64]. However, very few studies have tested -20°C conditions [65–67], though others employed these conditions for storing fecal samples and even soil samples for downstream microbial analyses [68–74]. For any study, post-collection biases can arise from many factors, including timing to transport samples, temperatures, and DNA preservation solutions [67, 75]. Nonetheless, two recent studies have determined that when all samples are stored in the same manner, the variability between samples prevailed over variability due to storage effects [65, 66]. Moreover, when specifically considering -80°C storage to seven other combinations of storage temperatures and DNA preservatives, the samples stored at -20°C and -80°C were most similar [66]. Clearly, for purposes of comparing studies, the -80°C storage conditions are most optimal. Thus, we acknowledge that our samples were stored at -20°C. Furthermore, when considering any studies characterizing microbiotas, it is essential that researchers examine the methods and metadata associated with those studies. Our comparisons with LEfSe considering individual features, as opposed to comparing multiple features at once, were more effective for understanding differences. It is likely that more complex and stratified studies would require many more horses and samples to better resolve relationships. Moreover, for specific management differences mentioned, when considering the effects they might have on a microbiota, it is necessary to have controlled studies with matching considerations for as many parameters as possible, even for conditions like matching diets, temporal range of events of parturition, and exposure to weather and precipitation.

## Conclusions

By using next-generation sequencing of the V4 domain of fecal DNA from foals at Days 1, 7, 28, 60, and weaning, we found that bacterial population compositions followed a pattern throughout the early life of the foal in an age-dependent manner. Moreover, we were also able to recognize differences in microbial populations amongst diarrheic foals, and we were able to detect microbial population differences that suggest impacts from differences in management styles between the facilities caring for these foals, though more thorough studies are required. Our future efforts will include strategies to better discern the effects of less abundant bacterial populations that may be just as important to GI health. Knowledge of how associations between microbial population profiles and animal management strategies can be used to inform horse owners and facility managers on the effects of their decisions on GI health of their horse herds.

## Supporting information

S1 Fig Principal Component Analysis Segregating Out farms. When considering beta diversity (unweighted unifrac), an examination of where each farm segregates within the PCA shows how samples by farm are represented within the cluster.
(PDF)

S1 Table Mare and foal set information. (XLSX)

S2 Table Farm management information. (XLSX)

S3 Table QIIME alpha diversity comparison. (XLSX)

S4 Table Percentage composition of microbial populations, by family (L5). (XLSX)

S5 Table ST2—L3 CategorySTAMP (color gradient high to low, p<0.05). (XLSX)

S6 Table ST2—L3 STAMP significant categories (p<0.05). (XLSX)
