# Correction: Utilizing the fecal microbiota to understand foal gut transitions from birth to weaning

**DOI:** 10.1371/journal.pone.0217721

**Published:** 2019-05-28

**Authors:** The PLOS ONE Staff

The fifth author’s name is spelled incorrectly. The correct name is: Elenamarie O’Malley. The correct citation is: De La Torre U, Henderson JD, Furtado KL, Pedroja M, O’Malley E, Mora A, et al. (2019) Utilizing the fecal microbiota to understand foal gut transitions from birth to weaning. PLoS ONE 14(4): e0216211. https://doi.org/10.1371/journal.pone.0216211. The publisher apologizes for the error.
