# Supplementary material for: Utilizing the fecal microbiota to understand foal gut transitions from birth to weaning
Source: PLoS One. 2019 Apr 30;14(4):e0216211. doi: 10.1371/journal.pone.0216211 (PMC6490953; doi:10.1371/journal.pone.0216211)
Supplement: S1 Fig — When considering beta diversity (unweighted unifrac), an examination of where each farm segregates within the PCA shows how samples by farm are represented within the cluster. (PDF) [file pone.0216211.s001.pdf]

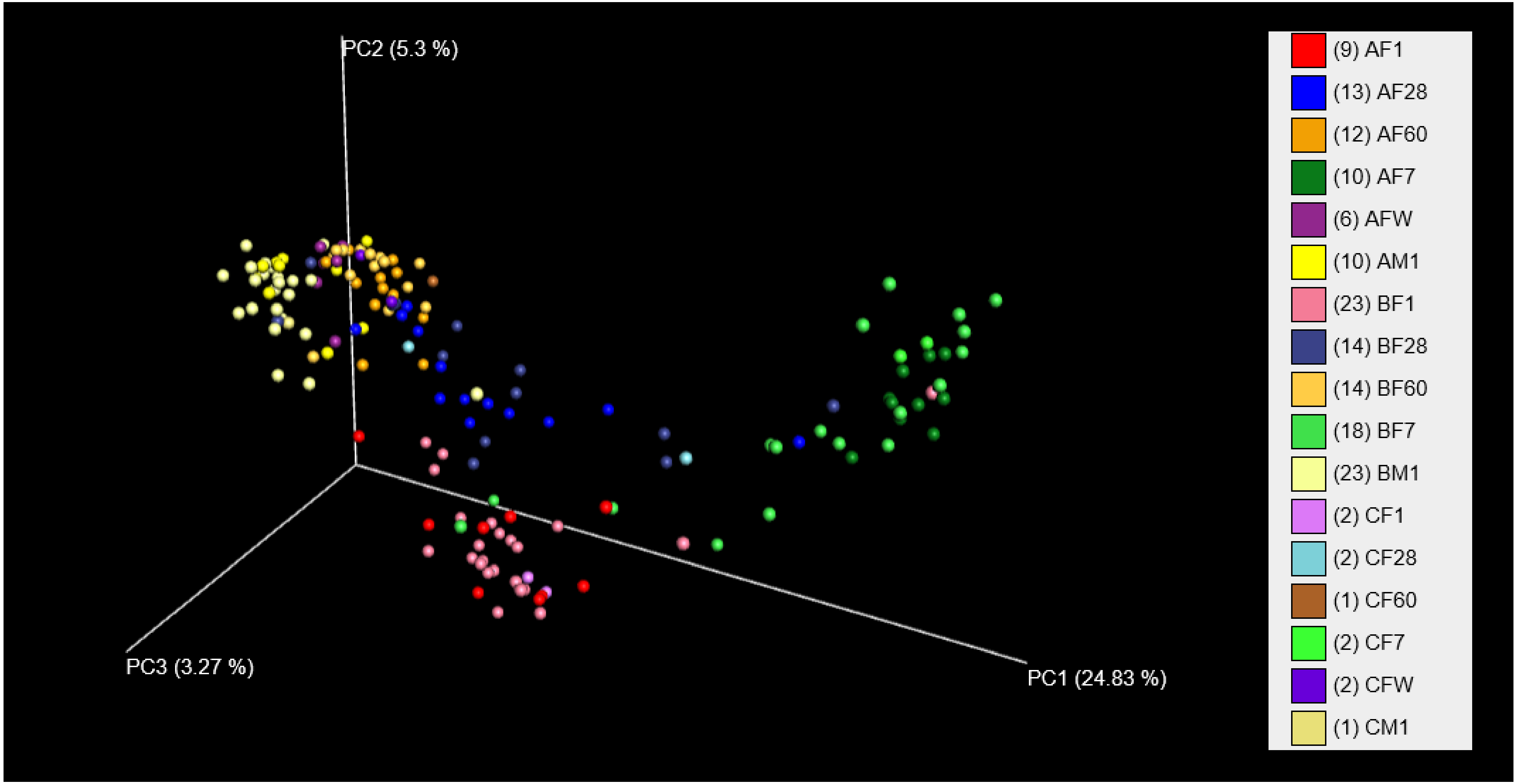

S1 Fig. Principal Component Analysis Segregating Out Farms. When considering beta diversity (unweighted unifrac), an examination of where each farm segregates within the PCA shows how samples by farm are represented within the cluster.
